# Effectiveness of E-Learning Program in Preventing WP Smoking in Adolescent Females in West of Iran by Applying Prototype-Willingness Model: A Randomized Controlled Trial

**DOI:** 10.34172/jrhs.2020.31

**Published:** 2020-11-05

**Authors:** Saeed Bashirian, Majid Barati, Manoochehr Karami, Behrooz Hamzeh, Elahe Ezati

**Affiliations:** ^1^Social Determinants of Health Research Center, Hamadan University of Medical Sciences, Hamadan, Iran; ^2^Research Center for Health Sciences, Hamadan University of Medical Sciences, Hamadan, Iran; ^3^Research Center for Environmental Determinacies of Health, School of Health, Kermanshah University of Medical Sciences, Kermanshah, Iran; ^4^Department of Public Health, School of Public Health, Hamadan University of Medical Sciences, Hamadan, Iran

**Keywords:** Adolescent, Female, Water pipe smoking

## Abstract

**Background:** Given the increasing trend of Water pipe (WP) smoking in adolescent females, it is necessary to use effective educational strategies in preventing WP smoking in developing countries. We aimed to determine effectiveness of e-learning program in preventing WP smoking in adolescent females west of Iran using prototype-willingness model.

**Study Design:** A randomized controlled trial.

**Methods:** This study was performed on 221 adolescent females in Kermanshah City, Iran during 2019-2020. Multistage random sampling was used. Data collection tool included a researcher-made questionnaire based on prototype-willingness model. E-learning-based intervention program included 5 training sessions. Participants were followed up for 3 months after the intervention. The data were analyzed using SPSS software.

**Results:** The mean scores of attitude, subjective norms, prototype, intention, and behavioral willingness structures were similar in both experimental and control groups before the educational intervention. However, after educational interventions, mean scores of structures of positive attitude towards WP, subjective norms about WP smoking, positive prototype about WP smokers, intention, and behavioral willingness towards WP smoking were decreased in the experimental group. Moreover, frequency of WP smoking was decreased in the experimental group compared to the control group after the educational intervention (*P*=0.003).

**Conclusion:** The use of e-learning-based interventions is an educational strategy for reducing WP smoking in adolescent females.

## Introduction

Tobacco epidemic is one of the biggest public health concerns in the world ^1^. Water pipe (WP) is one of the classic methods of tobacco use, which dates back to more than 400 years ago^2^. Prevalence of WP smoking in young population is rapidly increasing ^3^. In the past, WP smoking was common among men and was considered a fun and enjoyable tool. However, today, it has become popular among adolescents, especially females ^4^.

Communal nature of WP smoking often involves sharing of a single mouthpiece and hose between people, especially in social and communal settings. Besides, WP apparatus (including the hose and chamber) may contribute to this risk by providing an environment that promotes survival of microorganisms outside the body ^5^.

According to a comprehensive study, determinants of WP smoking in females include behavioral beliefs and attitudes^6-9^, subjective norms^7,10-12^, behavioral prototype ^13,14^, behavioral willingness ^7,13,14^ and behavioral intention ^15-18^.

The WHO has identified education as one of the most fundamental pillars of prevention programs ^19^. Based on the mentioned determinants, prototype-willingness model was selected as theoretical framework of this study. In this model, two types of decision-making are involved in health behaviors. These two types of decision-making offer two ways to engage in high-risk behaviors in adolescents: A) The path of logical action, in which adoption of behavior is an analytical process where the individual evaluates all aspects and then, takes action (such as the theory of reasoned action and planned behavior, B) The path of social reaction that is based on prototype and includes exploratory process for high-risk behaviors that adolescents engage in without prior intention and program ^20^.

According to this theory, in the path of logical action, the intention to perform a behavior is predicted by two factors: A) an attitude that is a positive or negative evaluation of a person's behavior; B) subjective norms that refer to social pressure perceived by the individual to do or not to do target behavior. Moreover, in the social reaction path of this model, behavioral willingness can be predicted by risk prototype structure ^21^. Comprehensive consideration of these studies showed that these interventions in the field of prevention of WP smoking were designed for target groups of students, young and adolescent boys ^22^ and the women; they have concentrated on review and qualitative studies ^23^. Therefore, according to prevalence of WP smoking among adolescent females and the lack of plan and intervention by related institutions, such as education, etc. and for addressing limitations of the studies on WP smoking in adolescents, it is necessary to design programs so as to prevent WP smoking in adolescents ^24^.

However, changes are taking place in the behavior and views of today's adolescents, most of which are influenced by introduction of new communication technology ^19^. Therefore, the present study was conducted to determine effectiveness of E-learning program in prevention of WP smoking in adolescent females west of Iran using prototype-willingness model.

## Methods

###  Study Design and Subjects

This randomized controlled trial was conducted among 221 middle and high school female students who were studying in four schools during 2019-2020 in Kermanshah city, western Iran. At first, a cross-sectional study was done in Kermanshah city to identify the schools with a high prevalence of WP smoking ^2^.

There was a 4-month interval between two phases of study (cross-sectional study and interventional study). Considering study power of 90%, type I error of 0.01 and also Mean ±SD of the behavior of reducing the WP smoking (27/57 ±5/60 and 25/86 ±6/5) ^25^, 108 students were selected as the study subjects. Regarding 10% attrition rate, sample size was calculated by 120 students for each group.

 Inclusion criteria for schools were as follows: public female middle and high school with a high prevalence of WP smoking located in Kermanshah City. In this study, 4 schools (2 schools in each grade) were randomly selected among the above-mentioned schools

 Eligible schools to participate in the study were selected by considering the average number of students in each class in high schools located in the study area and according to executive studies.

 Given similar demographic characteristics of schools (200 or 300 students), all the participants were randomly assigned (concealed from participants-single-blind) to two groups as experimental (n=2) and control (n=2). Totally, 120 participants were assigned to the experimental group (60 middle school students and 60 high school students) and there were 120 participants in the control group (similar to the experimental group). In each school, two classes were randomly selected to participate in the study.

 Nineteen students lost during follow-up period finally, data collection was carried out from 221 students (110 students in the experimental group and 111 students in the control group).

 Criteria for choosing the study participants included female high school students who had not been diagnosed with disability or mental and physical diseases and obtaining informed written consent from participants and their parents. Moreover, exclusion criteria were 2 times absence in training sessions and not being present in post-test.

###  Instrument 

 The data collection tool was a researcher-made questionnaire that was designed by a comprehensive review of research, literature, and the results of qualitative studies. The content validity of the questionnaire was evaluated by an expert panel consisted of 15 specialists in health education and promotion. The content validity ratio (CVR) and content validity index (CVI) was extracted. The internal consistency reliability was measured using Cronbach alpha. CVR, CVI, and Cronbach alpha coefficient of constructs. Obtained acceptable values: Attitude: α=0.76, CVR=0.81, CVI=0.93; Subjective Norms: α=0.86, CVR=0.83, CVI=0.96, Prototype: α=0.81, CVR=0.90, CVI=0.98, Behavioral Willingness: α=0.86, CVR=0.84, CVI=0.96; Intention: α= 0.86, CVR=0.86, CVI=0.96.

###  Demographic variables

 Demographic variables include age, degree of education, parental education, and parental occupation.

###  Water pipe smoking 

 In this section, questions related to WP smoking were presented, including experience of WP smoking during life (Yes or No), history of WP smoking at last month (Yes or No), place of first WP smoking and the first to be prepared for your WP.

The questions of the prototype-willingness model structures were performed using a comprehensive review of studies, especially studies of ^25-27^.

###  Positive attitude toward WP smoking 

 The questions related to individual attitudes toward WP smoking included 11 questions (for example, if I smoke WP, I can concentrate more) measured by a range of 5-point Likert answers from "strongly disagree" to "strongly agree". The scores of this structure ranged from 11 to 55. The highest score in this section indicated the positive attitude of students to WP.

###  Subjective norms about WP smoking 

 The questions related to abstract norms included 6 questions and the range of scores of this structure ranged from a minimum score of 6 to a maximum score of 30. Of the 6 questions, three were about the views of friends on WP, rated on a 5-point scale (I should not use at all, I must consume). The other three questions were about the views of friends about SS and their influence on the subject rated on a 5-point Likert scale from "always" to "never". The result of scores showed a positive view of friends about WP consumption, and their influence on subjects.

###  Prototype about WP smokers

 The questionnaire presented the prototype of a WP smoker and included 5 questions describing the person in terms of intelligence, inexperience, self-confidence, independence, and selfishness. The answers were rated on a 5-point Likert scale from "very much" to "never". A higher score indicated the positive prototype about WP smokers and prototype of a non- smoker included 5 questions describing the person in terms of intelligence, inexperience, self-confidence, independence, and selfishness. The answers were rated on a 5-point Likert scale from "very much" to "never". A higher score indicated the positive prototype towards non-smokers.

###  Behavioral willingness 

 The questionnaire of willingness to WP smoking includes a scenario in which adolescent thinks that she is among his friends engaged in WP and they persuade her to WP. In this section, the probability of the adolescent's reaction to this scenario was determined. The participants' probable reactions to suggestions about SS are reflected in 4 ways: a) I take the WP pipe and smoke one or more puffs; B) I smoke WP with my friends until the end; J) I thank them and I refuse to smoke; D) I leave that place. The answers were measured on a 5-point Likert scale ranging from "never" to "very much". The minimum and maximum scores were 4 and 20, respectively. A higher score indicated a greater willingness for WP smoking by a friend's suggestion.

###  Intention to WP smoking 

 There are 4 questions related to behavioral intention, which show the intention to WP smoking next month, next year, try not to use it for a lifetime, or decide not to go to places where WP is available (for example, "I may smoke a few puffs of WP in the next month"). The scores of this structure ranged from 4 to 20. A higher score in this questionnaire indicated a person's high intention to WP.

###  Educational intervention

 Designed educational intervention was implemented for the experimental group in 5 educational sessions according to analysis of pre-test results.

E-learning was used in the present study due to emphasis on the positive effect of using new educational methods in promoting health. The training program designed in this study was based on behavioral change strategies of web-based studies. In this regard, the results of a systematic study on behavioral change techniques showed that the most common techniques for behavioral change in web-based interventions were: A) Information approach by providing information about consequences of unhealthy behavior to learners and B) Skill training approach by identifying barriers to do a behavior, improving self-control and life skills, and reducing willingness to engage in high-risk behavior ^22^.

 For e-learning, the platform of the e-learning management system of Hamadan University of Medical Sciences was used. A personal page was designed for each participant in the e-learning management system.

 Then, for each person, user name and password were designed to enter their page. In the next step, the researcher taught the participants how to enter the system and personal page in person.

 After designing personal page, educational intervention consisting of educational materials, such as videos and an electronic training booklet was designed for the experimental groups for 5 wk educational session and was uploaded to the system for each participant.

**Figure 1:**
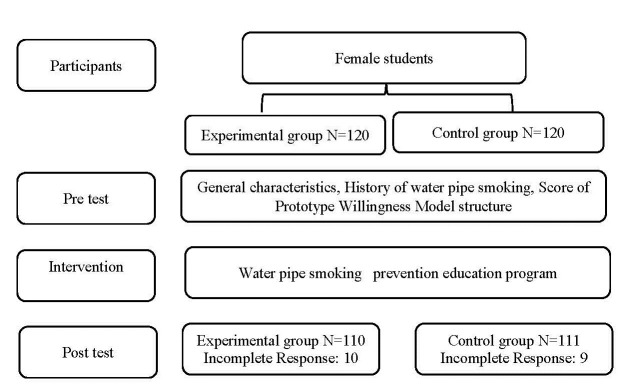
Study design

At the end of each training session, 3 questions were designed, and these questions were uploaded to the system. The purpose of designing the questions was that after watching educational video of each session and giving correct answers to the questions, the participants were able to go to the next clips. If answers to the questions were incorrect, they should watch tutorial clip again to identify correct answer. After 3 months of administrating the educational intervention, questionnaires were given to the participants in the study groups (Post-Test). The participants completed them by self-reporting way (figure 1).

###  Ethical consideration

 Written informed consent was obtained from students aged 16 yr and over and parents of students under 16 yr of age. The names of the participants in the questionnaire were not recorded and other information was kept confidential and used only for this study. The Ethics Committee of Hamadan University of Medical Sciences approved this study (reference number :(IR.UMSHA.REC.1397.696).

###  Statistical analysis

Statistical analysis data were analyzed by SPSS software (ver. 22 (Chicago, IL, USA), Wilco test (Comparison of mean score of structures in each group), Manvitny test (Comparison of the mean score of structures between groups), Chi-square test, MacNemar test in this study. *P*<0.05 was considered significant.

## Results

 In the pre-test stage, before the educational intervention, all the data of 240 female students were collected and considered. After the educational intervention and in the post-test phase, 19 subjects were rejected to continue the interventions (10 people in the experimental group and 9 people in the control group). The two groups who participated and were excluded from the study were not significantly different and the possibility of bias was limited.

Moreover, 35.9% of the participants in the experimental group and 35% of the participants in the control group had a history of WP smoking once during their lifetime, of which 51.2% of the experimental group and 64.3 of the control group for the first time had WP smoking prepared by their friends (Table 1).

Table 2 shows mean score of constructs including attitude, subjective norm, behavioral willingness, prototype, and behavioral intention at baseline and 3 months after the intervention. There was no significant difference in the prototype-willingness model constructs between the experimental and control groups at baseline.

Moreover, there was a significant decrease in the mean scores of positive attitude toward WP smoking (*P*=0.010), subjective norms about WP smoking (*P*=0.010), prototype about WP smokers (*P*=0.002), intention (*P*=0.002) and behavioral willingness (*P*=0.017) in the experimental group compared to the control group after the educational intervention (Table 2).

**Table 1:** Comparing the frequency of personal characteristics between intervention and control group Baseline (n=120)

**Characteristics**	Experimental group **Baseline (N =120)**		Control Group **Baseline (N =120)**		***P*** ** value**
	Frequency	Percentage	Frequency	Percentage	
Age (yr)					0.276
13-15	47	39.2	32	26.6	
15-17	45	37.5	58	48.3	
17-19	28	23.3	30	25.1	
High school grade ^a^		0.693
Eighth	31	25.8	30	25.0	
Ninth	31	25.8	28	23.3	
Tenth	30	25.2	32	26.7	
Twelfth	28	23.2	30	25.0	
Father's Education					0.158
Illiteracy	6	5.0	11	9.2	
Under the diploma	13	10.9	18	15.0	
Diploma	62	51.6	54	45.0	
College b	39	32.5	37	30.8	
Mother's Education					0.482
Illiteracy	9	7.5	12	10.0	
Under the diploma	26	21.6	31	25.8	
Diploma	64	53.4	53	44.2	
College b	21	17.5	24	20.0	
Father’s job^a^					0.650
Employee	38	31.6	29	24.2	
Self-employed	78	65.0	87	72.5	
Unemployed	4	3.4	4	3.4	
Mother’s job a					0.115
Housewife b	103	85.8	111	92.5	
Employed	10	8.4	7	5.8	
Self-employed	7	5.8	2	1.7	
The first place WP smoking (Former)					0.777
Home	10	23.2	10	23.8	
Family friend	13	30.3	17	40.4	
Park	6	13.9	7	16.6	
Cafe	14	32.6	7	16.6	
Wedding party	0	0.0	1	2.3	
How to prepared WP for the first time (Former)					0.627
Only Self	7	16.2	4	9.5	
Friend	22	51.2	27	64.3	
Family	9	20.9	10	23.8	
Relatives	5	11.7	1	2.4	
Water pipe smoking					0.439
Never	77	64.1	78	65.0	
Former	43	35.9	42	35.0	

**Figure 2:**
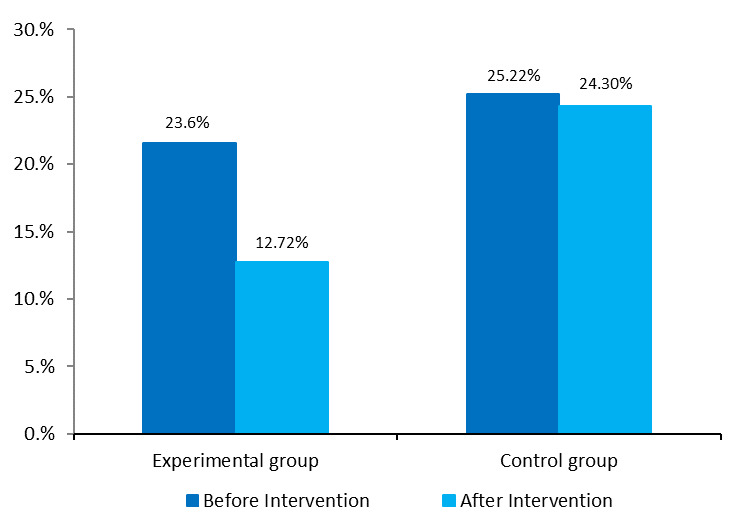
Comparing WP Smoking (last month) between Experimental and control group before and after the intervention

Figure 2 shows the results of considering behavior of WP smoking in the last month before and 3 months after the educational intervention in the experimental and control groups.

There was no significant difference in the frequency of WP smoking in the last month between the two experimental and control groups, before the intervention (*P*=0.784). However, after the educational intervention, frequency of WP smoking was significant in the last month between the experimental and control groups (*P*=0.020) (Figure 2). Results of the McNemar’s test showed a significant difference in frequency of WP smoking in the experimental group, in the last month before and after the educational intervention (*P*=0.003). In the control group, after the educational intervention, one case of decrease in the frequency of WP smoking was observed, which was not statistically significant.

**Table 2:** Comparison of mean scores variables before and 3 months after intervention in the control group and intervention group

**Groups**	**Before Intervention**		**After Intervention**		**Before and After Intervention**		***P*** ** value**
	**Mean**	**SE**	**Mean**	**SE**	**Mean**	**SE**	
Attitude							
Experimental group	25.64	12.30	17.06	9.70	-8.58	0.92	0.001
Control group	26.63	12.74	26.49	12.49	0.14	0.24	0.794
P value	0.751	0.010	
Subjective norms							
Experimental group	15.97	3.75	13.10	2.32	-2.87	0.35	0.001
Control group	16.10	3.22	15.96	3.33	0.14	0.10	0.177
P value	0.608	0.010	
Behavioral willingness							
Experimental group	9.79	5.21	8.49	4.64	-1.30	0.40	0.003
Control group	10.19	5.30	10.31	5.36	-0.12	0.08	0.097
P value	0.614	0.017	
Positive Prototype about WP Smokers							
Experimental group	14.74	3.76	12.73	3.95	-2.01	0.39	0.001
Control group	14.28	3.67	13.93	4.78	-0.35	0.13	0.824
P value	0.086	0.002	
Positive Prototype about non-WP person							
Experimental group	17,70	4.36	19.38	3.95	2.31	0.45	0.001
Control group	16.52	3.82	16.85	3.73	0.33	0.18	0.178
P value	0.205	0.004	
Behavioral intention							
Experimental group	10.10	4.87	8.90	5.46	-1.20	0.54	0.030
Control group	10.64	4.78	10.71	4.94	0.07	0.11	0.486
*P*-value	0.726	0.002			

## Discussion

 We aimed to determine effectiveness of e-learning program in preventing WP smoking in adolescent females west of Iran using prototype-willingness model.

Contrary to non-dynamic educational technologies such as printed texts, etc., e-learning allows a person to learn virtual reality, from what the person is supposed to learn by touching, etc. using tools, such as multimedia in the learning process, made this process more attractive and has changed peoples҆ attitude towards the lessons҆ subject ^28,29^.

Results of this study showed that the use of electronic educational intervention is an effective way in preventing WP smoking in adolescent females. This educational method seems to be cost-effective compared to face-to-face training ^30^.

E-learning provides access to training sessions at any time and place and eliminates unnecessary and time-consuming traffic to participate in training courses and avoids additional costs. Inviting a coach on the one hand and engaging of the individual in training and increasing self-confidence and attractiveness for people, especially adolescents, on the other hand, contributes to effectiveness of health-related interventions ^31^. Implementation and design of e-learning packages was effective in preventing smoking in adolescents^19^. Moreover, difference between average scores of the test were studied in the two groups of traditional and virtual education and the method of e-learning was more effective than traditional education, due to the presence of active and involved students in learning, deeper understanding of scientific content, and knowledge promotion in the students ^32^.

In a study on rural school students in west of England, level of creativity in students in Internet-based educational spaces increases dramatically and in fact, teaching in a traditional way encourages inclusive learning to become passive^33^. Moreover, the effect of education was compared in two ways: textbooks and educational clips in adolescents and showed that teaching by showing films and animations is more effective and motivating than the printed booklet, and they stated that educational clips and videos use the student's imagination due to colorful animations and teach them the issues in learning^34^. Using this approach, the students were practically involved and it created a kind of excitement and cooperation in the students, which can be the main factor in its effectiveness and motivation compared to the printed and written sources ^34^.

Besides, our findings showed a significant decrease in mean score of positive attitude towards WP smoking after the intervention in the experimental group compared to the control group indicating the effect of e-learning intervention on strengthening the negative attitude towards WP smoking in the experimental group. E-interventions led to strengthening the negative attitude towards smoking ^19,35^.

Tobacco prevention program (alcohol, cigarettes, WP, and other drugs) was effective in reducing the positive attitude towards tobacco ^36-38^.

Our results showed a significant difference in the mean scores of subjective norms encouraging WP smoking between the experimental and control groups after the intervention, i.e., after conducting the study, mean score of the subjective norms encouraging WP smoking was significantly reduced in the experimental group compared to the control group, which is consistent with the results of the previous studies ^39,40^.

The social learning theory, emphasizes interpersonal factors in explaining drug use. Adolescents acquire their beliefs about high-risk behaviors from role models, especially from close friends, relatives, and parents ^41^.

In this study, after the intervention, the positive prototypes of WP users had a significant decrease in the experimental group compared to the control group, while before the intervention; there was no significant difference between the experimental and control groups. Behavioral prototypes are considered as one of the most important assumptions of the prototype-willingness model, in which adolescents have clear social prototypes of the types of people of their age who engage in specific risky behaviors. Aspects of adolescents' prototypes of tobacco users (e.g., being independent and attractive) are exciting for teenagers. Therefore, one of the reasons that they start smoking is achieving some of these characteristics ^19,42^.

Intervening and correcting positive and desirable prototypes of smokers and strengthening their undesirable characteristics, or increasing attractiveness of prototypes of non-smokers can have a restraining and preventive effect on tobacco smoking ^20^.

The findings of the present study indicated that e-learning intervention reduced behavioral willingness for WP smoking among the participants in the experimental group, which is consistent with the results of the previous studies ^43^.

Web-based interventions were considered as one of the appropriate strategies to reduce tobacco smoking in adolescent boys ^19^.

Adolescents often find themselves in situations where they are encouraged to engage in risky behaviors, so they make irrational decisions. These decisions and the existence of these situations may be due to some environmental factors, such as easy access to cigarettes, WP, etc. Our findings also showed that mean score of intention to use WP was decreased significantly after the intervention in the experimental group compared to the control group, which is consistent with the prior studies ^44^.

In a group of people who were instructed about the harms of WP, about 62% of people had an intention to quit WP smoking ^45^.

 The intention increases by increasing level of willingness and behavioral willingness and intentions as the two predictors are appropriate for behavioral change.

In the experimental group, 32.5% of the participants had the first experience of WP smoking outside the home and in cafes and restaurants. The presence of WP in cafes and restaurants and access to WP for people under 18 yr of age has made the cafes an attractive and motivating place and a cheap way for young people to spend their free time ^23^.

Results of the present study showed that frequency of WP smoking was decreased significantly in the experimental group compared to the control group after the intervention, which is in line with the results of the previous studies ^40,25^.

## Conclusion

 The results of this study emphasize the need for e-learning interventions because of the cost-effectiveness and feasibility of implementing interventions. E-learning overcomes some of the barriers to face-to-face education and provides more flexibility for learning. It is also recommended that policymakers interventions reduce access to WP and enact laws to reduce access for smokers under the age of 18 to tobacco.

## Acknowledgements

 This study is a part of PhD thesis, approved by Hamadan University of Medical Sciences. Here, we are grateful to the Kermanshah city Instruction and Education authorities for their cooperation and support, school staff, and students that participated in this study.

## Conflict of interest

 All authors have no conflict of interest to declare.

## Funding

 This study has been supported by Hamadan University of Medical Sciences, Number grant (9612228251).

## Highlights

- Prototype-Willingness model could provide suitable intervention framework to preventing WP smoking in female adolescent students.
- E-learning-based interventions is an educational strategy for reducing WP smoking in students.
- Educational program based on Prototype-Willingness model had effects on positive attitude towards WP and behavioral willingness.
